# Bacillary angiomatosis mimicking squamous cell carcinoma in a patient with a history of chronic lymphocytic leukemia

**DOI:** 10.1016/j.jdcr.2025.07.022

**Published:** 2025-08-22

**Authors:** Svati Pazhyanur, Jihoon Baang, May P. Chan, Paul W. Harms, Kelly L. Harms

**Affiliations:** a University of Michigan Medical School, Ann Arbor, Michigan; b Division of Infectious Disease, Department of Internal Medicine, University of Michigan, Ann Arbor, Michigan; c Department of Pathology, University of Michigan, Ann Arbor, Michigan; d Department of Dermatology, University of Michigan, Ann Arbor, Michigan

**Keywords:** bacillary angiomatosis, clinical in-transit metastasis of squamous cell carcinoma, immunosuppression, squamous cell carcinoma

## Introduction

Bacillary angiomatosis, also known as epithelioid angiomatosis and cat scratch disease, is an uncommon disease caused by infection with *Bartonella henselae* or *Bartonella quintana*. It is characterized by neovascular proliferation in the skin or internal organs and presents with tumor-like masses. The most common clinical presentation is ≥1 papule(s) on the skin that develop into reddish to purple nodules, usually arising in the setting of immunosuppression.^1^ The nodules may grow as large as 10 cm in diameter. As a nodule enlarges, there is often central ulceration and bleeding. The most common location is the upper extremity.^2^

We report a case of bacillary angiomatosis presenting as a squamoproliferative lesion concerning for squamous cell carcinoma on the cheek of an immunosuppressed patient. This case highlights an uncommon clinical presentation of bacillary angiomatosis arising on the face associated with atypical squamoproliferative changes.

## Case report

**Fig 1:**
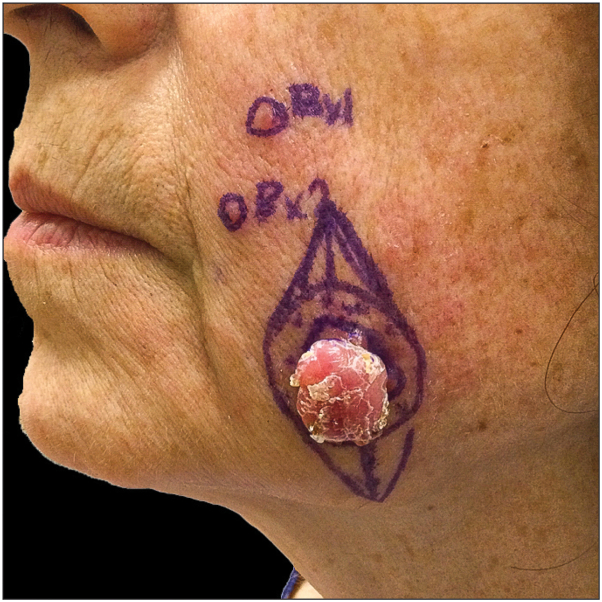
Polypoid and pedunculated nodule with scale on the cheek with adjacent smaller pink papules.

A 68-year-old female with untreated multiple sclerosis, untreated chronic lymphocytic leukemia (CLL), and hypogammaglobulinemia presented with a months-long history of a rapidly growing lesion on her left lower cheek. With trauma, the lesion had expressed clear fluid. Prior treatments included topical antibiotic treatment and a 5-day course of doxycycline with no improvement. The lesion was an erythematous papulonodule on the left lower cheek. Skin biopsy was interpreted as an atypical endophytic squamous proliferation adjacent to granulation tissue. Cluster of differentiation 31 and smooth muscle actin highlighted a nodular proliferation of capillary vessels in the papillary dermis. Tissue stains evaluating for bacteria, fungi, mycobacteria, and spirochetes were negative (Twort, Grocott-Gömöri methenamine silver, Fite acid fast, Ziehl-Neelsen, and Steiner). The differential diagnosis included a regressing keratoacanthoma, but the possibility of a more significant underlying process, such as squamous cell carcinoma, could not be excluded. The patient was referred for Mohs micrographic surgery. The lesion had grown to 1.5 cm in diameter, and 2 additional papules concerning for in-transit metastases were identified (Fig 1). Given the change in clinical presentation, further work up was recommended with excision of the original tumor-like lesion with formalin-fixed paraffin-embedded tissue processing and punch biopsies of both papules.

**Fig 2:**
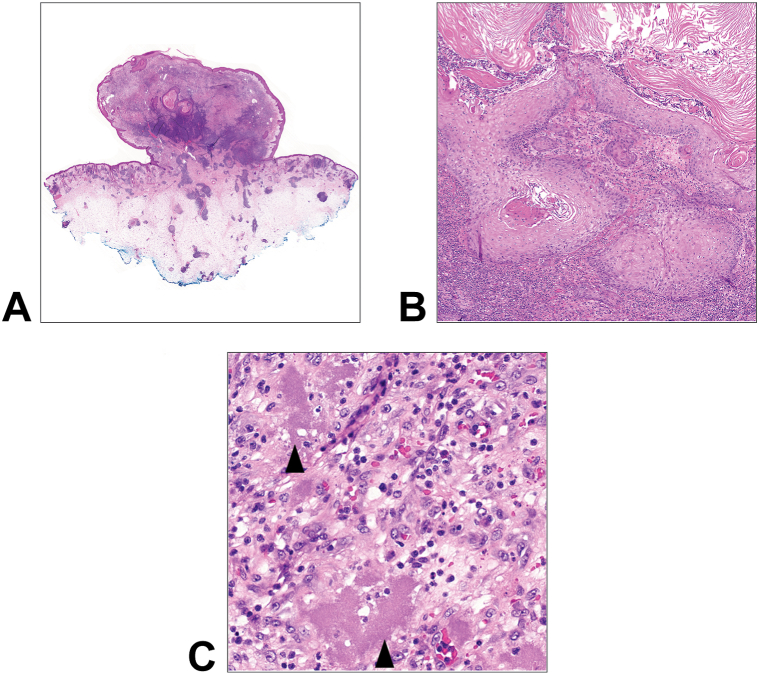
**A,** Polypoid lesion with cystic squamous proliferation. **B,** Squamous cystic structure with reactive changes. **C**, *Arrowheads* mark clumps of amphophilic granular material amid increased vessels. All stains are hematoxylin and eosin **(A, B,** and **C,** Hematoxylin-eosin stain; original magnifications: **A,** ×0.7; **B,** ×5; **C,** ×40**)**.

Pathology of the main lesion demonstrated a polypoid endophytic nodule with prominent well-formed vessels, aggregates of amphophilic granular material (Fig 2), zones of brisk neutrophilic inflammation, and peripheral aggregates of monotonous small lymphocytes consistent with recruitment of CLL. There were associated foci of pseudoepitheliomatous to cystic squamous hyperplasia (Fig 2). In the background dermis, there were scattered well-formed granulomata. CD34, ERG, and smooth muscle actin highlighted increased vessels, without epithelioid changes. No fungal, acid-fast, or bacterial organisms were identified. No definitive organisms were identified by Steiner stain. Despite the negative stains for microorganisms, the histopathologic features raised strong concern for an infectious process such as bacillary angiomatosis.

Broad-range tissue polymerase chain reaction (PCR) was performed on the tissue block and identified *Bartonella henselae*; additional bacterial, fungal, or mycobacterial organisms were not detected. The clinicopathologic findings were consistent with bacillary angiomatosis. Additional history revealed that the patient had been caring for several cats, including strays, that may have represented a source for *B. henselae*.

The patient was started on doxycycline and urgently referred to Infectious Disease. Because of immunosuppression, she was at risk for disseminated visceral disease and endocarditis. At that time, her symptoms included blurry vision, headaches, fatigue, and malaise. Workup with MRI of the brain and computed tomography of the chest, abdomen, and pelvis demonstrated abdominal lymphadenopathy and splenomegaly, and transthoracic echocardiogram revealed thickened aortic valve, which could not rule out endocarditis. *Bartonella* serologies were negative. Treatment was transitioned to rifampin and azithromycin due to lack of symptomatic improvement on doxycycline. She completed a 6-week course of rifampin and 12-week course of azithromycin, without evidence of relapse 4 months after completing antibiotics. During the course, immunoglobulin replacement therapy was initiated, and she also had progression of CLL requiring initiation of treatment.

## Discussion

This case of bacillary angiomatosis is notable for its atypical clinical presentation on the face and for the diagnostic challenge due to negative *Bartonella* serologies and rare histopathology demonstrating squamoproliferative changes.

The most common presentation of bacillary angiomatosis is a red-purple, vascular-appearing papule or nodule, usually on the upper extremities. Lymphadenopathy along the arm is also common in immunocompromised patients.^1^^,^^2^ The clinical differential diagnosis may include Kaposi sarcoma, pyogenic granuloma, cherry angioma, dermatofibroma, hemangioma, and mycobacterial infection such as tuberculosis, coccidioidomycosis, cryptococcosis, and histoplasmosis.^3^

Diagnosis of bacillary angiomatosis can be confirmed with histology and serologic testing. The characteristic histopathological features include a proliferation of well-developed engorged capillaries in an inflamed stroma with numerous inflammatory cells containing basophilic bacilli. Warthin-starry or Steiner stain will highlight clumps of bacteria, although these stains can be challenging to interpret. Serum testing for IgG and IgM of *Bartonella* species is more sensitive than culture methods; however, serologies have variable sensitivity and specificity and may be negative in patients with immunosuppression or HIV.^4^ In this case, hypogammaglobulinema likely contributed to the negative serologies. Blood, serum, or plasma PCR can also be used to differentiate between *Bartonella* species.^5^

Although bacillary angiomatosis most commonly involves the skin, it can also affect the bones and liver. Less commonly involved organs are the heart (endocarditis), brain, spleen, larynx, lymph nodes, and gastrointestinal tract. Symptoms of visceral involvement include weight loss, abdominal pain, and neuropsychiatric symptoms. Life-threatening complications include asphyxiation, and hepatic, splenic, or gastrointestinal hemorrhage. Workup for visceral organ involvement most commonly involves imaging of the abdomen with additional testing including MRI of the brain, computer tomography head or neck, and echocardiogram based on clinical suspicion.

Bacillary angiomatosis has been reported as mimicking pyogenic granuloma and Kaposi sarcoma.^6^^,^^7^ However, cases mimicking squamous cell carcinoma are exceedingly rare, with only 3 previously published cases demonstrating pseudoepitheliomatous hyperplasia.^8^, ^9^, ^10^ This case is uncommon due to the unusual histopathologic combination of squamous changes with granulomatous inflammation and demonstrates the role of tissue PCR to facilitate the diagnosis given negative serologies and lack of organisms identified with appropriate tissue staining. Correct diagnosis and distinction from squamous cell carcinoma are crucial to avoid unnecessary treatment such as surgical excision, as bacillary angiomatosis is typically managed with antibiotic therapy.

## Conflicts of interest

None disclosed.
